# Clinicopathological characteristics and postoperative prognosis of patients with nuclear pedigree of esophageal squamous cell carcinoma

**DOI:** 10.3389/fonc.2023.1190457

**Published:** 2023-07-19

**Authors:** Meng Xia Wei, Xin Song, Xue Ke Zhao, Wen Li Han, Qi De Bao, Xue Nan Han, Rui Hua Xu, Xin Min Li, Zong Min Fan, Ran Wang, Xing Song Li, Jing Feng Hu, Jia Li, Bei Li, Hui Fang Tan, She Gan Gao, Fu You Zhou, Li Dong Wang

**Affiliations:** ^1^ State Key Laboratory of Esophageal Cancer Prevention & Treatment, Zhengzhou University, Zhengzhou, China; ^2^ Henan Key Laboratory for Esophageal Cancer Research of The First Affiliated Hospital, Zhengzhou University, Zhengzhou, China; ^3^ Oncology Department, He’nan Puyang Municipal Any Ang Area Hospital, Anyang, China; ^4^ Department of Pathology, The Maternal and Child Health Care Hospital of Zhengzhou, Zhengzhou, Henan, China; ^5^ Department of Language, Zhengzhou White Gown Translation Co., Ltd., Zhengzhou, China; ^6^ Department of Gastroenterology, People’s Hospital of Zhengzhou, Zhengzhou, China; ^7^ Department of Oncology, the First Affiliated Hospital, Henan University of Science and Technology, Luoyang, China; ^8^ Oncology Prevention Office, Anyang Tumor Hospital, Anyang, China

**Keywords:** esophageal cancer, nuclear pedigree, clinicopathological characteristics, prognosis, COX analysis

## Abstract

The aim of this work is to analyze the clinicopathological characteristics and prognostic factors of patients with nuclear pedigree of esophageal cancer. The clinicopathological data and follow-up information of 3,260 patients from different nuclear pedigree of esophageal cancer who underwent radical resection of esophageal cancer were collected, and the clinicopathological characteristics and prognostic factors of the patients were analyzed. The male to female ratio of 3,260 patients with esophageal cancer was 1.7:1. The diagnosis age was ranged from 32 to 85 (60.2 ± 8.1) years old. About 53.8% of the patients were ≥ 60 years old; About 88.8% of the patients came from the high incidence area of esophageal cancer; About 82.5% of the tumors were located in the middle and lower segments of esophagus; Poor, moderate and well differentiation accounted for 26.6%, 61.9% and 11.5% respectively; The surgical margin accounted for 94.3%; 47.6% of the tumors were shorter than 4 cm in length; Clinicopathological TNM stage (0+I) accounted for 15.2%, and stage II, III and IV accounted for 54.5%, 29.9% and 0.4%, respectively. Cox analysis showed that male, diagnosed age ≥ 60 years, tumor located in neck and upper esophageal segments, poor differentiation, tumor length ≥ 4 cm, and advanced TNM were independent risk factors for the prognosis of patients in nuclear pedigree with esophageal cancer. Gender, diagnosis age, tumor location, degree of differentiation, tumor length and TNM stage are the influencing factors for the prognosis of patients with nuclear pedigree of esophageal cancer, which will provide important data for the future study of esophageal cancer family aggregation.

## 1 Introduction

China is one of the countries with the highest incidence rate and mortality rate of esophageal cancer, and nearly half of the new esophageal cancer in the world occurs in China (1, 2). The obvious familial aggregation is a prominent epidemiological feature of esophageal cancer in China, which is manifested by the fact that more than one patient in a family suffer from esophageal cancer. It is reported in literature that among the seven generations of a family, there are 96 patients with esophageal cancer (3). In the past studies, high risk families were often used to express the phenomenon of family aggregation of esophageal cancer (4, 5). Because the nuclear pedigree of esophageal cancer definition standard was not clear, it was inconvenient to use it.

Based on the 500,000 sample library of esophageal cancer in the State Key Laboratory of Esophageal Cancer Prevention & Treatment and Henan Key Laboratory for Esophageal Cancer Research of The First Affiliated Hospital, Zhengzhou University, Zhengzhou, Henan Province. We firstly proposed the definition of the nuclear pedigree of esophageal cancer.

**Figure 1:**
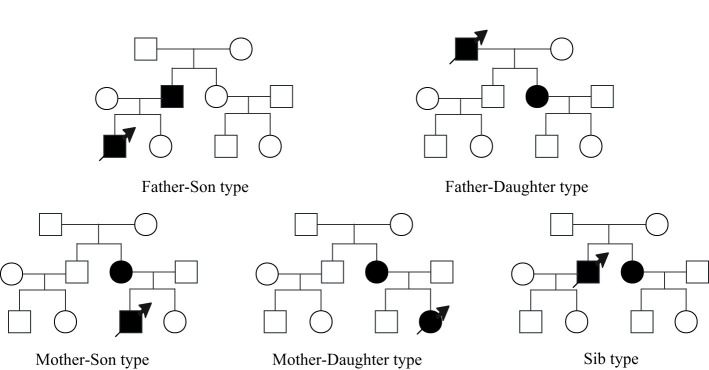
Schematic diagram of nuclear pedigree of esophageal cancer.

The nuclear pedigree of esophageal cancer is defined as a family with ≥ 2 patients of esophageal cancer in two generations of family members (4, 6, 7). This classification encompasses five distinct types, including to father-son type (father and at least one son suffer from esophageal cancer), father-daughter type (father and at least one daughter suffer from esophageal cancer), mother-son type (mother and at least one son suffer from esophageal cancer), mother-daughter type (mother and at least one daughter suffer from esophageal cancer) and sibling-type (siblings suffer from esophageal cancer together), as shown in **Figure 1**. Nuclear pedigree represents a specific form of family history associated with esophageal cancer. However, due to the long time interval between the onset of esophageal cancer in different generations of patients, it is difficult for the proband to obtain detailed clinical diagnosis and treatment information of his father or mother after being found, moreover, it is more difficult to collect complete esophageal biopsy and (or) postoperative tissue samples of all patients, which leads to a shortage of valuable research data on esophageal cancer of nuclear pedigree. This is the main reason for the slow progress of relevant research in this field.

Eepidemiological investigations of esophageal cancer show that esophageal cancer has obvious family aggregation characteristics (8). Numerous studies have demonstrated a significant increase in the risk of esophageal cancer when an individual’s first degree relatives are affected by esophageal cancer (4, 9), and the more esophageal cancer patients in the first degree relatives, the greater the risk, suggesting that genetic factors play an important role in the occurrence and development of esophageal cancer. As a typical representative of the phenomenon of family aggregation of esophageal cancer, patients in the nuclear pedigree of esophageal cancer have the characteristics of high tumor susceptibility and high incidence rate, and play an irreplaceable role in the research of molecular mechanism of esophageal cancer. In the past, a large number of studies have analyzed the factors affecting the prognosis of patients with esophageal cancer, but few reports have been made on the molecular indicators affecting the prognosis of patients with esophageal cancer in nuclear pedigree families.

This paper analyzes the clinicopathological characteristics and prognostic factors of patients in the nuclear pedigree families of esophageal cancer for the first time, providing scientific basis for clinical evaluation, early prevention and prognosis improvement of patients in the nuclear pedigree family. Additionally, the nuclear pedigree family of esophageal cancer is the best model for identification and localization of esophageal cancer susceptibility genes, as well as driving candidate genes. Therefore, it is of great clinical value to analyze the molecular indicators affecting their prognosis.

## 2 Research participates and method

### 2.1 Research participates

This research was enrolled 3,260 patients of esophageal cancer collected from the clinical information database of 500,000 sample library of esophageal cancer in the State Key Laboratory of Esophageal Cancer Prevention & Treatment and Henan Key Laboratory for Esophageal Cancer Research of The First Affiliated Hospital, Zhengzhou University, Zhengzhou, Henan Province.

Inclusion criteria of this study were as follows: ① All patients were hospitalized in Anyang Cancer Hospital, and the clinical and pathological information was complete. ② All patients received radical resection of esophageal cancer. ③ Pathological diagnosis confirmed as esophageal squamous cell carcinoma. ④ All patients come from different nuclear pedigree. Exclusion criteria were as follows: ① Radiotherapy and/or chemotherapy were received before or after operation. ② Complicated with other malignant tumors (10). The clinical and pathological characteristics of this study include gender, age of diagnosis, high and low incidence areas, tumor location, tumor length, differentiation, surgical margin, tumor invasion depth, lymph node metastasis and distant organ metastasis. Refer to the Clinicopathological TNM staging of esophageal cancer of the 7th edition of the American Joint Commission on Cancer (AJCC), and combine with the postoperative pathological report to determine the TNM staging of patients. Patients information is provided in **Table 1**.

**Table 1:** Clinicopathological characteristics of 3,260 patients with nuclear pedigree of esophageal cancer.

Clinicopathological characteristics	Cases	Percentage (%)
Gender		
Male	2043	62.7
Female	1217	37.3
Diagnosis age (years old)		
<60	1505	46.2
≥60	1755	53.8
High/Low incidence area		
High incidence area	2896	88.8
Low incidence area	364	11.2
Tumor site		
Neck+Upper	486	17.5
Middle+Lower	2774	82.5
Differentiation degree		
Poor differentiation	867	26.6
Moderate differentiation	2017	61.9
Well differentiation	376	11.5
Edge condition		
Negative	3075	94.3
Positive	185	5.7
Tumor length (cm)		
<4	1552	47.6
≥4	1708	52.4
Clinicopathological TNM stage		
0+I stage	494	15.2
II stage	1776	54.5
III stage	976	29.9
IV stage	14	0.4

### 2.2 Follow-up

The forms of survival follow-up mainly include the following:

① Check the medical record information, obtain the phone number of the patients or the contact person, and conduct telephone or SMS follow-up; ② Contact the corresponding township health center or village health center according to the detailed home address registered in the medical record information; ③ Take household visits survey and on-site follow-up.

From the date of diagnosis, follow-up was conducted by telephone or household survey, with death as the end point. The patient will be followed up every 3 months in the first year after discharge, and once a year thereafter. The deadline is December 2018.

### 2.3 Statistical analysis

SPSS 21.0 was used for analysis. Kaplan Meier method was used to draw survival curves of patients with different clinical characteristics and log rank test was performed; Cox regression model was used to screen the independent factors affecting the prognosis of patients in the nuclear pedigree of esophageal cancer. Inspection level α = 0.05.

## 3 Result

### 3.1 Clinicopathological characteristics of the 3,260 patients

Of the 3,260 patients in the nuclear pedigree of esophageal cancer, 2,043 were male (62.7%) and 1,217 were female (37.3%), with a male to female ratio of 1.7 ∶ 1; The age of diagnosis was 32∼85 (60.2 ± 8.1) years old, and 1,755 cases (53.8%) were ≥ 60 years old; 2,896 cases (88.8%) came from the high incidence area of esophageal cancer; 486 cases (17.5%) were located in the neck and upper segments of the esophagus, 2,774 cases (82.5%) were located in the middle and lower segments of the esophagus; 867 cases were poorly differentiated (26.6%), 2,017 cases were moderately differentiated (61.9%), and 376 cases were well differentiated (11.5%); 3,075 cases (94.3%) had a negative margin; 1,552 cases (47.6%) with tumor length<4 cm; TNM stage 0+I 494 cases (15.2%), II 1,776 cases (54.5%), III 976 cases (29.9%), IV 14 cases (0.4%). The clinicopathological information were showed in **Table 1**.

### 3.2 Influencing factors of postoperative prognosis

#### 3.2.1 Univariate analysis

**Figure 2:**
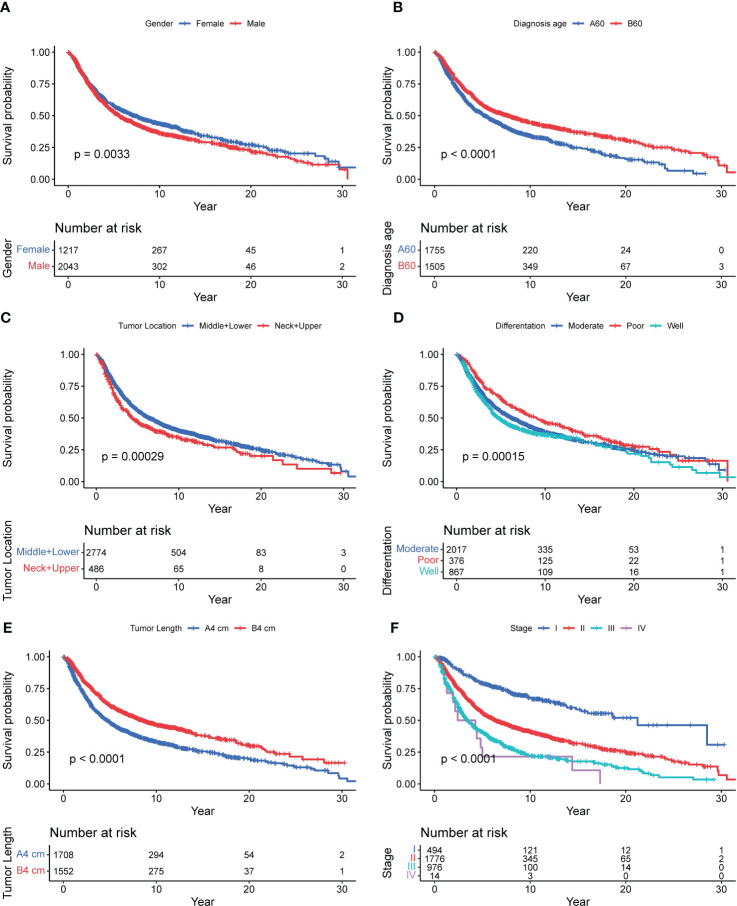
Survival curve of 3,260 patients with nuclear pedigree of esophageal cancer. [**(A)**: Gender; **(B)**: Diagnosis age; **(C)**: Tumor location; **(D)**: Difference degree; **(E)**: Tumor Length; **(F)**: Stage].

Kaplan Meier survival analysis and log rank test of 3,260 patients with esophageal squamous cell carcinoma showed that the overall median survival period of 3,260 patients was 6.1 (2.2, 19.3). The median survival time of males was 5.6 (2.2, 18.0), and that of females was 7.3 (2.4, 21.8), 7.7 (2.7, 24.8) years old for <60 years old and 5.2 (2.0, 14.5) years for ≥ 60 years old; Patients in high incidence areas were 6.0 (2.2, 19.3), and patients in low incidence areas were 7.0 (2.4, 20.1); Tumor location: 4.3 (1.8, 16.9) for neck+upper segment, 6.4 (2.4, 19.9) for middle+lower segment; Differentiation degree: 4.7 (1.9, 18.5) for poor differentiation, 6.2 (2.3, 19.9) for moderate differentiation, 9.1 (3.1, 22.8) for well differentiation; Surgical margin: 6.0 (2.2, 19.1) for negative, 6.4 (2.3, 21.7) for positive; Tumor length: 8.5 (3.0, 22.8) for <4 cm, 4.7 (1.9, 15.5) for ≥ 4 cm; Clinicopathological TNM stages: 21.2 (7.0, –) for 0+ I stage, 6.4 (2.5, 19.1) for II stage, 3.4 (1.6, 8.8) for III stage, and 2.4 (1.3, 5.0) for IV stage, and the results were shown in **Table 2**. Univariate analysis showed that gender (χ^2 ^= 8.637, *P*=0.003), age of diagnosis (χ2 = 41.951, *P*<0.001), tumor location (χ^2 ^= 13.139, *P*<0.001), differentiation (χ^2 ^= 17.562, *P*<0.001), tumor length (χ^2 ^= 74.526, *P*<0.001) and clinicopathological TNM stage (χ^2 ^= 255.862, *P*<0.001) were related to the prognosis of patients in the core family of esophageal cancer. And the results were showed in **Table 3** and **Figure 2**.

**Table 2:** Variable assignment.

Variables	Assignment
Gender	0 = Female; 1 = Male
Diagnosis age	0 =“<60 years old”; 1 =“≥60 years old”
Tumor site	0 = Neck and upper segment; 1 = Middle and lower sections
Differentiation degree	0 = Well differentiation; 1 = Moderate differentiation; 2 = Poor differentiation
Tumor length	0 =“<4 cm”; 1 =“≥4 cm”
Clinicopathological TNM stage	0 = (0 + I) stage; 1 = II stage; 2 = III stage; 3 = IV stage

**Table 3:** Comparison of K-M survival curves of patients with esophageal cancer in nuclear pedigree families with different clinicopathological characteristics.

Clinicopathological characteristics	*n*	MST (T75, T25)/a	χ2	*P*
Diagnosis age(years old)				
<60	1,505	7.7(2.7, 24.8)	41.951	<0.001
≥60	1,755	5.2(2.0, 14.5)		
Gender				
Male	2,043	5.6(2.2, 18.0)	8.637	0.003
Female	1,217	7.3(2.4, 21.8)		
Tumor length				
<4 cm	1,552	8.5(3.0, 22.8)	74.526	<0.001
≥4 cm	1,708	4.7(1.9, 15.5)		
High/ Low area				
High incidence area	2,896	6.0(2.2, 19.3)	0.596	0.440
Low incidence area	364	7.0(2.4, 20.1)		
Tumor site				
Neck+Upper	486	4.3(1.8, 16.9)	13.139	<0.001
Middle+Lower	2,774	6.4(2.4, 19.9)		
Differentiation degree				
Well differentiation	867	9.1(3.1, 22.8)	17.562	<0.001
Moderate differentiation	2,017	6.2(2.3, 19.9)		
Poor differentiation	376	4.7(1.9, 18.5)		
Surgical margin				
Negative	3,075	6.0(2.2, 19.1)	0.004	0.950
Positive	185	6.4(2.3, 21.7)		
Clinicopathological TNM stage				
0+I stage	494	21.2(7.0, -)	255.862	<0.001
II stage	1,776	6.4(2.5, 19.1)		
III stage	976	3.4(1.6, 8.8)		
IV stage	14	2.4(1.3, 5.0)		

MST, Median survival time.

The high incidence areas of esophageal cancer was higher than 50/100,000, and the low incidence areas of esophageal cancer was lower than 50/100,000 (11).

#### 3.2.2 Multivariate analysis

**Figure 3:**
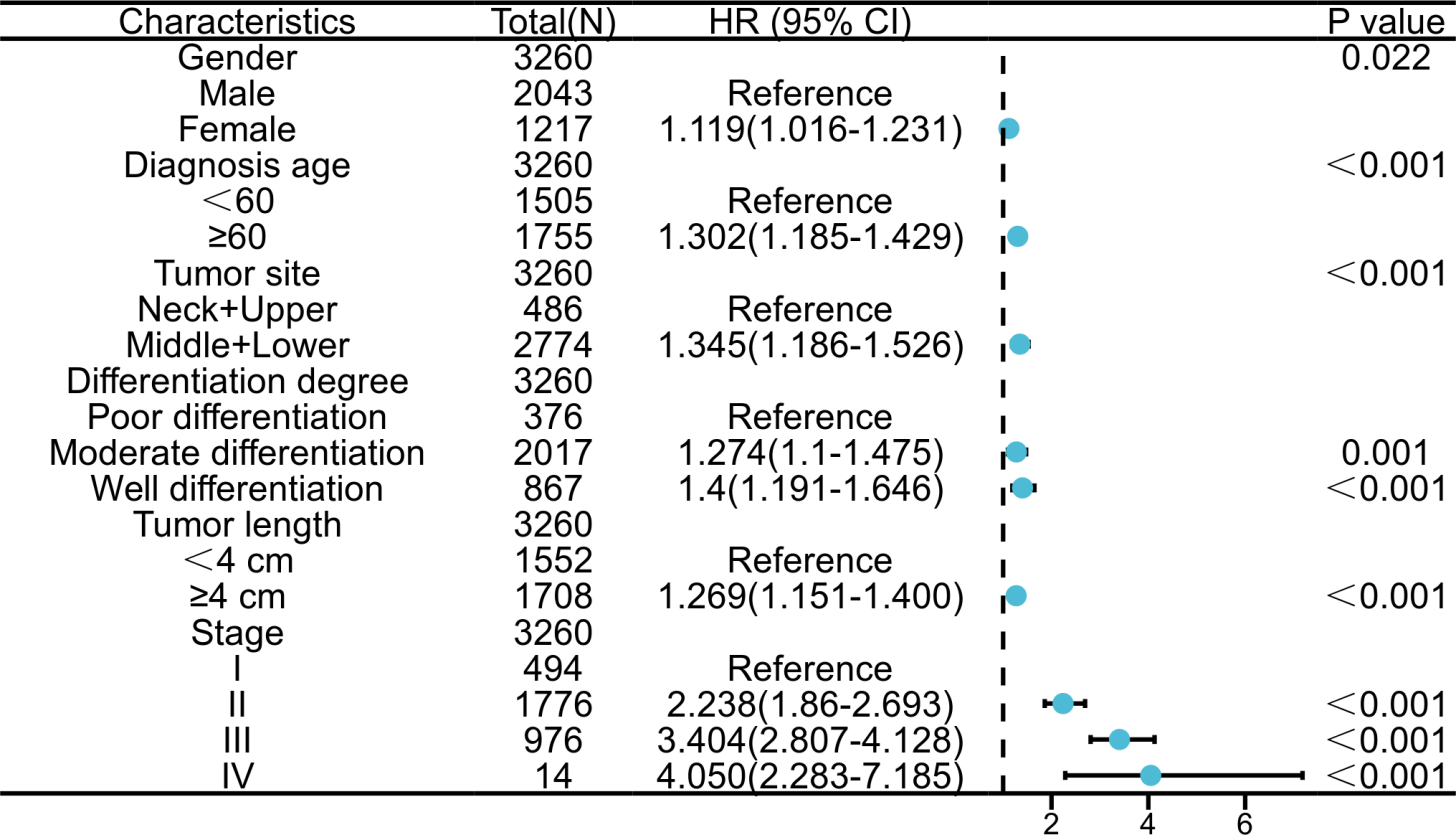
Forest diagram for 3,260 patients with nuclear pedigree of esophageal cancer.

**Figure 4:**
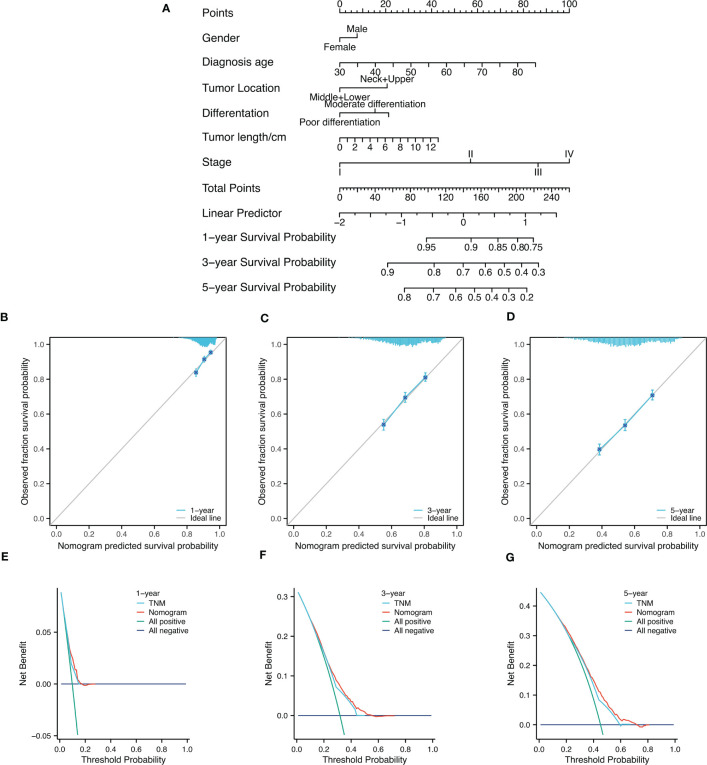
Nomogram diagram analysis. **(A)** Nomogram for survival of patients with progressive patients with nuclear pedigree of ESCC. **(B-D)**1-year, 3-year, and 5-year nomogram calibration curves to predict survival probability in ESCC; **(E-G)** DCA chart showed a certain advantage in predicting 3-year and 5-year OS for nomogram compared with AJCC-TNM staging system.

The analysis of collinearity diagnosis on gender, diagnosis age, tumor location, differentiation degree, tumor length and clinicopathological TNM stage shows that there is no collinearity, and the tolerance are 0.995, 0.998, 0.995, 0.993, and 0.988, respectively. The VIF values are 1.005, 1.002, 1.005, 1.007, and 1.012, respectively. The factors with statistical significance in the single factor analysis were included in the multivariate analysis. **Table 2** were the variable assignment. The results showed male (HR=1.119, 95%CI=1.016-1.231, *P*=0.022), diagnosis age≥60 (HR=1.302, 95%CI=1.185-1.429, *P*<0.001), neck+upper (HR=1.345, 95%CI=1.186-1.526, *P*<0.001), poor differentiation (HR=1.149, 95%CI=1.065-1.238, *P*<0.001 =, Tumor length≥4cm (HR=1.269, 95%CI=1.151-1.400, *P*<0.001) and advanced TNM (HR=1.666, 95%CI=1.547-1.795, *P*<0.001) are independent risk factors affecting the prognosis of patients in nuclear pedigree families with esophageal cancer, as shown in **Table 4**. Multivariate Cox analysis (nomogram) results showed that gender, diagnosis age, tumor location, differentiation degree, tumor length, and stage are independent predictor of overall survival (*P*<0.05) (**Figure 3**). A nomogram using the risk factors obtained from multivariate Cox regression analysis was constructed to predict the 1-, 3-, and 5-year overall survival of patients with ESCC (**Figure 4A**). Each factor has its corresponding score value on the points scale and the maximum score is 100 points. A total score could be obtained by adding the scores of all selected factors, and then the corresponding 1-, 3-, and 5-year overall survival could be estimated by the nomogram scoring system. The nomogram revealed that stage was the most influential prognostic factor. In addition, diagnosis age made a moderate contribution to the survival outcome, while tumor length, gender, tumor location, and differentation played minor roles (**Figures 4B–D**). The DCA showed that the nomogram had good clinical value (**Figures 4E–G**).

**Table 4:** Cox regression analysis.

Variables	*β*	*SE*	*P*	*HR*(95%*CI*)
Gender	0.112	0.049	0.022	1.119(1.016~1.231)
Diagnosis age	0.264	0.048	<0.001	1.302(1.185~1.429)
Tumor site	0.297	0.064	<0.001	1.345(1.186~1.526)
Differentiation degree	0.139	0.038	<0.001	1.149(1.065~1.238)
Tumor length	0.239	0.050	<0.001	1.269(1.151~1.400)
Clinicopathological TNM stage	0.511	0.038	<0.001	1.666(1.547~1.795)

#### 3.2.3 Family situation

Among 3,260 nuclear pedigree with esophageal cancer, the proband was father had 1,534 patients of esophageal cancer, mother had 1,388 patients of esophageal cancer, both parents had 344 patients of esophageal cancer, and compatriots had 1,044 patients of esophageal cancer. There were 2,527 families of 2 patients with esophageal cancer, 583 families of 3 patients with esophageal cancer, and 150 families of more than 4 (including 4) patients with esophageal cancer; There were up to 8 patients of esophageal cancer in a single nuclear pedigree.

## 4 Discussion

As a special family aggregation phenomenon, the nuclear pedigree family is helpful to strengthen our understanding of the family aggregation phenomenon of esophageal cancer, and it showed very important clinical significance. The cause of the nuclear pedigree phenomenon is not very clear at present. Li (12)found that the highest frequency of esophageal cancer among direct relatives within three generations was 70% through the investigation of cancer clustering families. Some scholars (13) have investigated the pedigrees of patients with esophageal cancer, and found that the closer the blood relationship with the patients, the higher incidence rate of esophageal cancer, indicating that genetic factor play an important role in the occurrence of esophageal cancer in the nuclear pedigree. Diet, living habits, and the same environmental exposure factors may be the reasons for the clustering of ESCC in families.

It also has been reported that the different mechanisms underlying tumorigenesis in patients with ESCC with and without a family history of the disease remained, and in ESCC patients with familial ESCC show germline mutations in *BAX*, *CDKN2A*, *TP53*, and *CHEK1*, and tumor-specific mutated genes in patients with a positive family history of ESCC were *APC*, *AKT3*, *DPYD*, *EP300*, *NFE2L2*, *PPP2R1A*, *RUNX1*, and *VEGFA* (14). Germline mutation of *BRCA2* may play a role in familial aggregation of ESCC (15), but we found no reports discussed the germline mutation difference between ESCC with and without nuclear pedigree. And a strong cumulative effect of familial ESCC specific genetic loci for ESCC risk, and eight SNPs (rs12917, rs7206735, rs12947788, rs4785204, rs1042522, rs2238149, rs353163 and rs647126) showed a significant association with ESCC among individuals with a family history of esophageal cancer (16). Patients with familial ESCC harbored more mutations in gene coding regions within ESCC tumors (14).

This study found that male, and diagnosed age ≥ 60 years old were the risk factors for the prognosis of patients with esophageal cancer in the nuclear pedigree, which was consistent with the previous analysis of the prognosis of patients with esophageal cancer (17, 18). The function of organs in elderly patients is reduced, and their immunity is reduced, and they are often accompanied by various chronic diseases. Their overall physiological condition is obviously inferior to that of young patients. Clinicians tend to be conservative in their choice of diagnosis and treatment plans for elderly patients, which will also affect the survival time of patients to a certain extent (19). Gender, as an independent influencing factor of patients in the nuclear pedigree, may be related to male patients exposed to more risk factors. It is reported (20) that most men have bad habits such as smoking and drinking, high work pressure, irregular diet and sleep, while women pay more attention to good diet and living habits. Similarity results have been revealed by Hou (21), and well differentiation showed the best prognosis, and then moderate, poor differentiation showed the worst prognosis. From **Figure 2C**, we observed that middle+lower tumor location showed better prognosis than neck and upper (*P*<0.001), which was agree with Dong’s result (22) unless statistically not significant (*P*=0.146). Tumor length and TNM stage reflect the depth of invasion of tumor, and regional lymph node metastasis, the later the stage, the larger tumor, the deeper the tumor invasion, the more extensive the regional lymph node metastasis, and the cells in the center of the tumor are less sensitive to radiotherapy and therefore have poor prognosis (11, 23).

According to the results of this study, the occurrence of tumors in the neck and upper segments of the esophagus is a risk factor for the prognosis of patients in the nuclear pedigree of esophageal cancer. Because of the proximal location of the lesion, it is difficult to obtain a safe surgical margin, and total laryngectomy during neck surgery cannot be accepted by most people, so it is relatively difficult to resect neck and upper esophageal cancer (24). Some studies (25) showed that the resection rate and 5-year survival rate of upper esophageal cancer were significantly lower than those of lower esophageal cancer. The degree of tumor differentiation also affects the prognosis of patients in the nuclear pedigree of esophageal cancer. The malignant degree of tumor is related to the degree of differentiation. The lower the degree of differentiation, the higher the degree of malignancy, and the greater the possibility of lymph node metastasis, leading to a better prognosis (26).

This study also found that tumor length affected the prognosis of patients with esophageal cancer in the nuclear pedigree. Through a series of studies, and others of our research group (27–29)confirmed that the tumor length is highly related to the degree of invasion and lymph node metastasis of esophageal cancer. With the increase of tumor length, the degree of invasion gradually deepens, and the rate of lymph node metastasis also increases, which will directly affect the prognosis of patients in nuclear pedigree of esophageal cancer. Clinicopathological TNM staging is also an independent factor affecting the postoperative prognosis of patients in the nuclear pedigree of esophageal cancer, which suggests that the prognosis of patients in the nuclear pedigree families of esophageal cancer can be predicted according to the TNM staging standard formulated by AJCC, which has guiding significance for the selection of clinical diagnosis and treatment programs.

In conclusion, this study analyzed the clinicopathological characteristics of patients in the nuclear pedigree of esophageal cancer and the influencing factors of postoperative prognosis, which will provide important data for the future study of esophageal cancer family aggregation.

## Data availability statement

The original contributions presented in the study are included in the article/supplementary material. Further inquiries can be directed to the corresponding author.

## Author contributions

MW wrote the manuscript, and HT, RX, XZ, WH, XH, BL, and XS provided the data analysis help, SG, QB, and FZ providing the clinical information, JL provided the linguistic services, and LW designed the research. All authors contributed to the article and approved the submitted version.
